# A Case Based Approach to Clinical Genetics of Thoracic Aortic Aneurysm/Dissection

**DOI:** 10.1155/2016/9579654

**Published:** 2016-05-25

**Authors:** Betti Giusti, Stefano Nistri, Elena Sticchi, Rosina De Cario, Rosanna Abbate, Gian Franco Gensini, Guglielmina Pepe

**Affiliations:** ^1^Department of Experimental and Clinical Medicine, Section of Critical Medical Care and Medical Specialities, DENOTHE Center, University of Florence, Florence 50134, Italy; ^2^Department of Heart and Vessels, Marfan Syndrome and Related Disorders Regional Referral Center, Careggi Hospital, Florence 50134, Italy; ^3^Cardiology Service, CMSR Veneto Medica, Altavilla Vicentina, Vicenza 36077, Italy; ^4^S. Maria agli Ulivi Center, Fondazione Don Carlo Gnocchi, Onlus, IRCCS, Florence 50023, Italy

## Abstract

Thoracic aortic aneurysm/dissection (TAAD) is a potential lethal condition with a rising incidence. This condition may occur sporadically; nevertheless, it displays familial clustering in >20% of the cases. Family history confers a six- to twentyfold increased risk of TAAD and has to be considered in the identification and evaluation of patients needing an adequate clinical follow-up. Familial TAAD recognizes a number of potential etiologies with a significant genetic heterogeneity, in either syndromic or nonsyndromic forms of the manifestation. The clinical impact and the management of patients with TAAD differ according to the syndromic and nonsyndromic forms of the manifestation. The clinical management of TAAD patients varies, depending on the different forms. Starting from the description of patient history, in this paper, we summarized the state of the art concerning assessment of clinical/genetic profile and therapeutic management of TAAD patients.

## 1. Introduction

Thoracic aortic aneurysm/dissection (TAAD) is a potential lethal condition with a rising incidence and its clinical impact, as well as the therapeutic management, differs according to the syndromic or nonsyndromic manifestation. Thus, the understanding of the family history together with a comprehensive clinical and genetic evaluation of patients with TAAD is necessary for clinical diagnosis in order to achieve a documented differential diagnosis and therapeutic medical and surgical strategies. Starting from a real-world clinical case report and referring to the state of the art in the field, this review aims to summarize the principal diagnostic phases with the clinical/genetics evaluations allowing the achievement of the most appropriate management of TAAD patients.

## 2. Clinical Relevance of Family History and Screening Strategies

**Figure 1:**
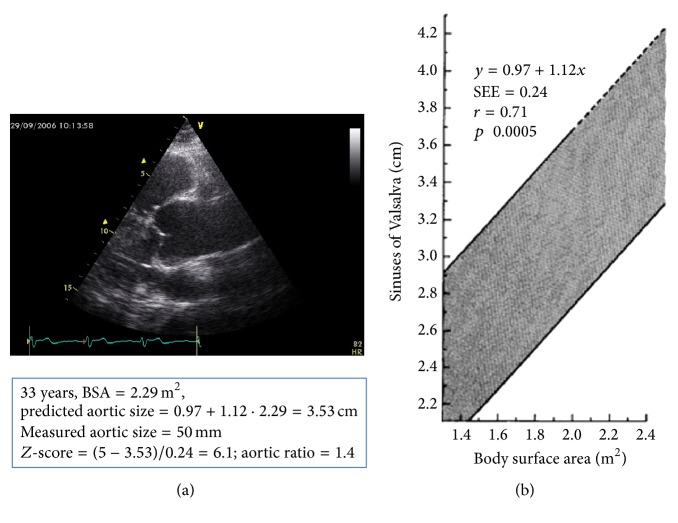
(a) Parasternal long-axis view of the aortic root and of proximal ascending aorta of our patient, at end-diastole. (b) 95% confidence limits for aortic root diameter at the sinuses of Valsalva in relation to body surface area in adults younger than 40 years (modified from Roman et al. 1989 [1]). In the box, based on the nomogram, predicted size of the aortic root is calculated (3.53 cm) and compared with measured aortic size (5.0 cm) to calculate the *Z*-score and the aortic ratio.

**Figure 2:**
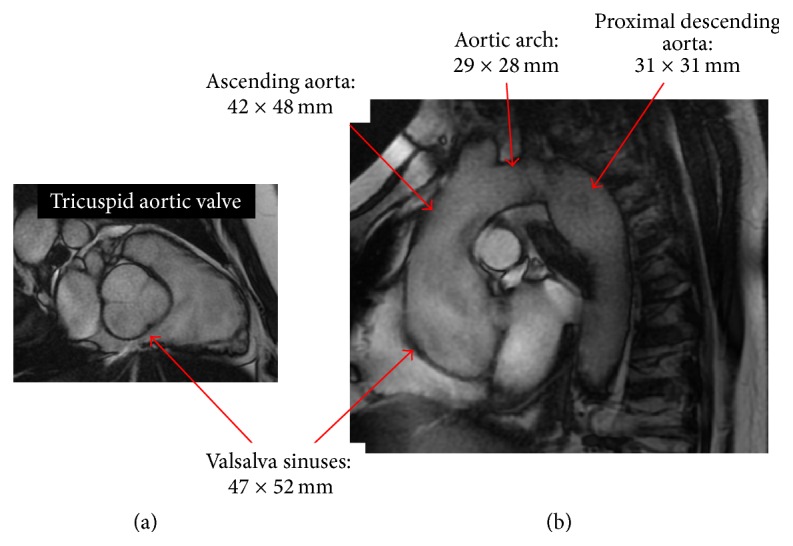
Magnetic resonance SSFP-based imaging: (a) a diastolic sinus plane image, showing 3 aortic cusps; (b) SSFP sagittal oblique of the ascending aorta's aortic arch and proximal descending aorta. Reported measurements were all performed by orthogonal views at end-diastole.

In September 2006, a 33-year-old male (height 193 cm, weight 98 kg, and body surface area 2.29 m^2^) was referred by his general practitioner to a private cardiologist for the evaluation of systemic arterial hypertension, treated with ramipril 2.5 mg o.i.d. and amlodipine 5 mg o.i.d. Family history was positive for fatal type A aortic dissection occurring at 52 years in the patient's mother. The patient had undergone surgery for inguinal hernia in 2000 and reported hiatal hernia and myopia >3 diopters. At cardiovascular evaluation, blood pressure was 138/90 mmHg; resting ECG showed sinus rhythm with 74 beats per minute and incomplete right bundle branch block. Echocardiography evidenced aortic root ectasia (50 mm; *Z*-score 6.1, aortic ratio (RR) 1.4) and dilatation of the proximal ascending aorta (42 mm) (Figure 1) associated with a mildly regurgitant tricuspid aortic valve and with a trivial mitral regurgitation. The left ventricle was normal in size and function. In order to achieve a full evaluation of the entire aorta, the patient was submitted to cardiovascular magnetic resonance (Figure 2) which showed severe dilatation of the aortic root (47 × 51 mm), diffuse dilatation of the ascending aorta (maximal diameter 42 × 48 mm), and mild dilatation of the aortic arch and of the proximal descending aorta (31 × 31 mm). Therapy was changed into metoprolol 25 mg t.i.d. (with the suggestion to increase the dosage up to reach target heart rate < 60 bpm) and amlodipine 5 mg o.i.d.

Thoracic aortic aneurysm/dissection (TAAD) is a potential lethal condition with a rising incidence [2]. Although this condition may occur sporadically, it displays familial clustering in >20% of the cases [3–5]. In particular, family history confers a significant increased (six- to 20-fold) relative risk of TAAD which is not conveyed by cardiopulmonary comorbidity [6]. A positive family history is known to represent a risk factor for the development of TAAD and can be considered in the evaluation and identification of patients at increased risk [7–9]. Evidences exist indicating that between 15% and 30% of patients with an aortic aneurysm and/or dissection have a positive family history of TAAD risk [7–9]. Thus, an echocardiographic screening of first-degree relatives of patients with TAAD, as well as in relatives of individuals affected by conditions in which thoracic aneurysm and dissection are common clinical features of the disease (e.g., Marfan syndrome (MFS), Loeys-Dietz syndrome (LDS), and bicuspid aortic valve (BAV)), is recommended to favor early detection and to establish appropriate follow-up and therapeutic strategies [10]. Noteworthily, such a screening policy was not performed in the herein described patient, who underwent echocardiography for the assessment of potential organ damage due to arterial hypertension.

Familial TAAD predominantly displays an autosomal dominant pattern of inheritance, with varying degrees of penetrance and variable expressivity, although other forms of inheritance, including recessive patterns, have been reported [7–9]. In comparison to sporadic TAAD patients, familial TAAD individuals tend to be younger at presentation, suggesting a more aggressive clinical phenotype [6]. Beyond a positive family history of TAAD, criteria for diagnosing nonsyndromic familial TAAD include (a) detection of dilatation at any trait of the thoracic aorta involving either the sinuses of Valsalva, the ascending aorta, or both and (b) the exclusion of MFS, LDS, Ehlers-Danlos syndrome vascular type, and other syndromic causes of TAAD (Table 1).

Transthoracic echocardiography allows adequate assessment of several aortic segments, particularly the aortic root and proximal ascending aorta. All scanning planes should be applied to obtain information on most aortic segments [12]. Particular care should be also used to visualize the morphology of the aortic valve, since BAV is frequently associated with dilatation either of the ascending aorta distal to the sinuses of Valsalva or of the sinuses of Valsalva [13] and may be a complex familial trait [14]. The diagnosis of dilatation of the sinuses of Valsalva or of proximal ascending aorta is based on the comparison of measured aortic diameter compared with age-appropriate nomograms related to body surface area (BSA) [1]. Consistently, an aneurysm of the thoracic aorta (as well as of any other arterial region) is defined as a permanent localized dilatation having at least a 50% increase in diameter compared with the expected normal size [15]. Thus, the ratio between the measured aortic size and the expected aortic diameter (so-called aortic ratio (RR)) should be >1.5 to appropriately allow the definition of aneurysm. Aortic dilatation exceeding the expected one by <150% is called ectasia/dilatation. Moreover, subtracting the measured aortic size from the expected aortic diameter and dividing this number by the standard deviation provided by the nomogram will supply the aortic *Z*-score (Figure 1).

When inconclusive information or abnormalities (i.e., dilatation ≥ 40 mm) are present in the echocardiographic examination, another imaging modality is required (namely, computed tomography (CT) scan or magnetic resonance (MR)) to either complete or add diagnostic assessment [12]. These methods, in fact, allow full visualization on the entire aorta, independently of the quality of the acoustic windows, potentially affecting transthoracic echocardiography. However, for both diagnostic and follow-up purposes, the utilization of CT and MRI appropriate planes and standardized lines of measurements are mandatory to reduce misclassifications and subsequently inappropriate therapeutic choices [16].

## 3. Etiopathogenetic Profile of Thoracic Aortic Aneurysm/Dissection and Therapeutic Implications: The Role of Tertiary Referral Centers

The patient was referred to the Center for the Study of Aortopathies, Marfan Syndrome, and Related Disorders of Florence University, for clinical evaluation, genetic counseling, and, eventually, genetic analysis. At clinical examination, the patient displayed enophthalmos, malar hypoplasia, retrognathia, pectus carinatum, dorsolumbar scoliosis, reduced elbow extension, hindfoot deformity, and skin striae. With the above-described clinical manifestations, the patient was reclassified as affected by MFS. Thus, we started therapy with metoprolol 25 mg t.i.d. and losartan (starting from 50 mg o.i.d. to reach optimal therapy) and suggested aortic surgery. The patient was subsequently evaluated by the cardiac surgeon of his country's hospital, who decided to accept him for surgery pending the result of genetic assessment.

Familial TAAD recognizes a number of potential etiologies with a significant genetic heterogeneity (Table 1), in either syndromic or nonsyndromic forms of the manifestation. Both forms may include sporadic cases due to “novel/fresh” mutations. In fact, many genes have been associated with TAAD and many other potential genes have been localized on various chromosome regions [4, 5].

TAAD is often part of the clinical manifestations of pleiotropic inherited connective tissue disorders, with the principal being MFS. MFS displays an autosomal dominant inheritance, with a prevalence of 2 per 10,000. Mutations in* FBN1* gene encoding fibrillin 1 have been described in 70–90% of patients fulfilling MFS diagnosis [11]. Actually, literature data showed that patients exhibiting suspected MF phenotype not harbouring* FBN1* mutations were carrier of mutations in other relevant genes mainly* TGFBR1* and* TGFBR2* coding transforming growth factor receptor types 1 and 2 (<3%) (prevalently associated with LDSs) [11]. Clinical criteria for MFS diagnosis are represented by TAAD, ectopia lentis, and systemic features with a score ≥7 (Tables 2 and 3). Presence of a first-degree relative affected by MFS and the detection of a pathogenic mutation in* FBN1* gene are other two criteria (Table 2). The presence of at least two criteria (two clinical or one clinical and one genetic) allows the diagnosis of MFS according to the revised Ghent criteria [11] (Table 2). The correct diagnosis of MFS requires a* multidisciplinary team* relying on a set of diagnostic criteria which include two different road maps according to the presence of family history (Table 2), in association with a systemic score describing the presence of systemic involvement of the disease (Table 3).

In our patient, the clinical manifestations are sufficient for making diagnosis of MFS with the presence of aortic ectasia (*Z*-score > 2) and systemic features (score = 8: enophthalmos + malar hypoplasia + retrognathia (1), pectus carinatum (2), dorsolumbar scoliosis (1), reduced elbow extension (1), hindfoot deformity (2), and skin striae (1)) as criteria (Table 3).

The diagnosis of MFS has multiple implications for a patient, given the more aggressive pattern of the disease in comparison with other potential etiologies. The implications regard the need for specific medical treatment, the counseling for follow-up, pregnancy and family planning, and advice for aortic surgery, which are indicated at lower threshold of aortic size in MFS than in non-MFS patients [17].

A number of studies have assessed the potential role of multiple therapies in MFS. The pathophysiology of aortopathy in MFS constitutes the basis for the use of *β*-blockers, originally proposed to decrease rate of rise of aortic pressure, although this effect has not been proven in patients with major aortic dilatation [18–23]. The beneficial effect of *β*-blockade may also be due to the bradycardia-induced decrease of the rate of stretching of the aortic wall, as well as to decrease in blood pressure, although the hypotensive effect of *β*-blockers has never been evaluated in a randomized study in normotensive individuals. Similarly, the utilization of a nondihydropyridinic calcium-channel antagonist in case of intolerance to *β*-blockers is reasonable. Consistently, the avoidance of stressful, intense physical and sport activities should be a part of clinical counseling in MFS patients. Recently, the effectiveness of such a policy in preventing aortic dissection in a great proportion of MFS patients has been shown in 732 patients who were followed up for a mean of 6.6 years [24], further supporting the notion that *β*-blockers are recommended in MFS patients. The novel interpretation of the pathophysiology of MFS-related aortopathy based on TGF-*β* signaling [25] has resulted in researches evaluating the potential of TGF-*β*-antagonism by ACE-inhibitors and angiotensin II receptor blockers (ARB) [21, 22, 26]. Groenink et al. (2013) [27] have recently reported the results of a prospective, randomized, controlled trial indicating a beneficial effect of losartan treatment on aortic root dilatation rate in adults with MFS. Furthermore, Mueller et al., in a retrospective cohort of previously untreated pediatric patients with MFS, demonstrated that both strategies with *β*-blockers or ARB are beneficial in pediatric and adolescent patients [26].

Since then, multiple studies explored this issue with somehow conflicting results. Lacro et al., among children and young adults with Marfan's syndrome who were randomly assigned to losartan or atenolol, disappointingly found no significant difference in the rate of aortic root dilatation between the two treatment groups over a 3-year period [28]. Moreover, the Marfan Sartan trial showed that the evolution of the size of the aortic root was not modified by adding losartan to *β*-blocker therapy, discouraging the utilization of ARB as first-line therapy in these patients [29]. Furthermore, a recent randomized, double-blind trial using MRI to compare the efficacy of losartan and atenolol, given as monotherapy, did not demonstrate differences between treatments in their efficacy to prevent aortic dilation in MFS [30]. Finally, a recent experimental publication argued for a treatment strategy that would target both promiscuous angiotensin receptors and TGF-*β* signaling without interfering with the early protective role of TGF-*β* activity [31]. Differences among the various study designs, including doses and combination of various drugs, and differences in imaging modality, as well as the characteristics of the enrolled patients, preclude, anyway, a definite statement regarding medical treatment in MFS. Overall, the results of the available studies raise concern on the real effectiveness of any medical treatment for the overall population of MFS patients, possibly due to their wide clinical and genetic heterogeneity. Indeed, MFS is often seen as a single disease in clinical studies but in MFS different types of mutations in the same gene as well as mutations in different genes are involved. Recent data suggested that Marfan patients with haploinsufficient FBN1 mutations seem to be more responsive to losartan therapy for inhibition of aortic root dilatation rate compared with dominant negative patients [32]. We are now interpreting the results from studies on patients with similar clinical picture but different genetic mechanisms. While future researches and planned individual meta-analysis [33] could shed some light on such discrepancies among studies, the approach on the single patient basis should be tailored and adjusted based on a strict clinical, genetic, and instrumental evaluation.

Based on the available data at the moment in which we assessed the patient, aiming at reducing heart rate at <70 bpm and at lowering blood pressure, we withhold amlodipine and choose to associate 100 mg of losartan with 25 mg of metoprolol t.i.d.

The diagnosis of MFS also affects indications to prophylactic aortic surgery since death from aortic dissection or rupture occurs in untreated MFS patients often before 40 years, and dilation of the aortic root is present in 60–80% of patients [24–27, 34]. In patients with sporadic TAA without major connective tissue disease, aortic surgery is recommended when maximal aortic size is ≥55 mm, provided that BAV or ≥5 mm/year of increase in aortic size is not shown or that another cardiac surgery is indicated [17]. In individuals with MFS, aortic surgery is otherwise recommended when aortic size is ≥50 mm, or ≥45 mm when other risk factors are present. These risk factors include family history of aortic dissection, rapid increase in aortic size, severe aortic and/or mitral regurgitation, and desire of pregnancy in women [17]. Patients with TAA, independently of the etiology, should be reassessed on a periodic basis both clinically and with appropriate imaging modalities. Behind evaluation of aortic dilatation, including exploration of symptoms suggestive of TAA expansion, comprehensive quantitative evaluation of both aortic and mitral hemodynamic derangement is needed. Moreover, stringent control of hypertension, smoking cessation (and avoidance of exposure to environmental tobacco smoke), lipid profile optimization (based on risk profile), and other atherosclerosis risk-reduction measures should be instituted for patients with TAA of any size and etiology, independently of the indication to surgery. Even if a poor or complete lack of association of traditional cardiovascular risk factors with aortic root aneurysm in syndromic and nonsyndromic disease was observed, the increase in the life expectancy of the affected patients raises the need to protect these patients from other atherosclerotic comorbidities and from the deleterious effect of atherosclerosis on a “fragile” aortic wall due to alteration of the extracellular matrix and extracellular matrix remodeling [35]. Finally, patients should be educated for prompt self-referral to hospital facilities in presence of chest or back pain.

Therefore, due to (1) the diagnosis of MFS, (2) the aortic diameter of 50 mm at Valsalva sinus, and (3) the positive family history for aortic dissection, our patient was referred to aortic surgery, independently of genetic analysis, which does not play any role for the indication on timing of surgery in this patient in whom diagnosis has been fully accomplished clinically.

Indeed, mutation screening analysis should be applied to the cases in which clinical features are not sufficient to reach a final diagnosis [11], for prenatal diagnosis in couples at risk, or in families in which mortality associated with aortic aneurysms/dissections along generations is present. In the latter case, the identification of a pathogenetic mutation may help in performing prevention and careful follow-up in younger relatives at risk.

## 4. Genetic Approaches

Nonetheless, the patient underwent genetic analysis in the genetic laboratory of the Center for the Study of Aortopathies, Marfan Syndrome, and Related Disorders of Florence University for the identification of pathogenetic mutation, after genetic counseling, due to the need expressed by the patient for future prenatal diagnosis. A written informed consent was obtained from the patient.

The genetic analysis for the identification of the pathogenetic mutation in MFS consists of the analysis of the three major MFS associated genes:* FBN1*,* TGFBR2,* and* TGFBR1* [11]. The analysis workflow starts with the analysis of the* FBN1* gene detectable in 70–90% of MFS patients [11, 35–37], and then, in the case of negative result, it is extended to* TGFBR2* and* TGFBR1* gene.

**Figure 3:**
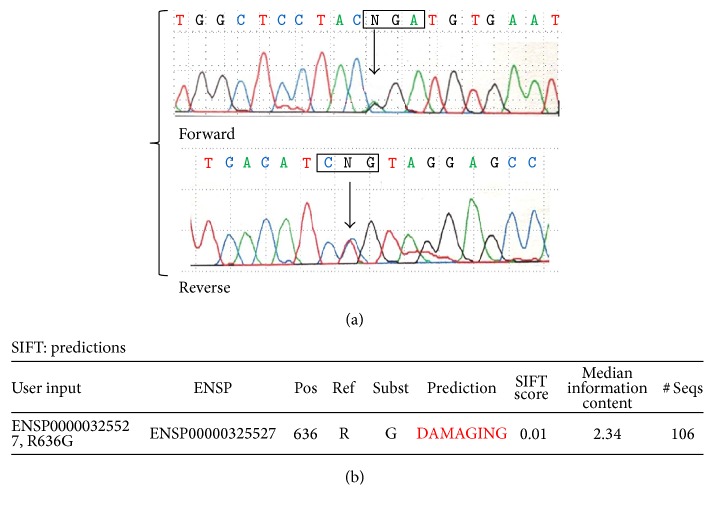
Chromatogram details of the forward and reverse sequencing of* FBN1* gene exon 15 and flanking intronic regions. The sequence (a) shows a pathogenetic missense mutation (c.1906 A>G, p. Arg636Gly) as evaluated by SIFT* in silico* prediction (b).

Despite the technological advances, the genetic analysis is still time consuming and relatively expensive and most importantly requires an expert genetic laboratory. The classical genetic analysis approach consists of direct sequencing of the 65 coding exons and intronic flanking regions of* FBN1* gene, followed in case of negative result by sequencing of* TGFBR2* (7 exons) and then* TGFBR1* (9 exons) coding and flanking regions by Sanger technology. The direct sequencing of exons including the intron junction sequences is crucial to allow the identification of mRNA splicing defects. Once the putative pathogenetic mutation has been identified, the genetic laboratory needs to evaluate the “real pathogenicity” according to criteria reported in Table 2 and/or by using* in silico* tools such as SIFT (http://sift.jcvi.org/), Polyphen (http://genetics.bwh.harvard.edu/pph2/), and Pmut (http://mmb.pcb.ub.es/PMut/).  Figure 3 shows chromatogram details of an example of FBN1 sequencing analysis and* in silico* effect of the identified mutation.

In our patient, the genetic analysis of the three major MFS genes did not evidence the pathogenetic mutation. This finding did not override either the previous clinical diagnosis of MFS or the presence of a mutation in the three major genes, due to several potential explanations: presence of (1) a large insertion/deletion involving several exons or the entire candidate genes (FBN1 or TGFBR2 or TGFBR1) [38–42] and (2) mosaicism in the three major genes [42–48]. These types of mutations, already reported in some Marfan syndrome patients by the literature, are not identified by the Sanger sequencing approach or require DNA analysis from more than one kind of cell (fibroblast, lymphocytes, salivary cells, or gonadal cells) [38–51]. Finally, a further explanation might be the presence of pathogenetic mutations in genes at present unknown to be associated with MFS.

On the other hand, syndromes caused by mutations in the TGF-*β*-signaling system, causing different forms of Loeys-Dietz syndrome, can share clinical features with Marfan syndrome [11]. In these disorders, the genes that might be mutated include TGFBR1, TGFBR2, TGFB2, TGFB3, and SMAD3 [52–59]. This patient, at the moment, does not show any other feature suggestive for TGF-*β*-pathies such as Loeys-Dietz syndrome [52–59].

Both in the case of pathogenetic mutation identification and in the case that no pathogenetic mutation is identified, the genetic counseling is mandatory to explain the implication for the investigated subject and for the family members, especially when the aim of the genetic test is the possibility of prenatal diagnosis. The genetic counseling should be performed according to the current legislation of the specific country.

The development and standardization of high-throughput sequencing technologies (HTS) or next-generation sequencing (NGS) also for diagnostic purpose are determining a lowering of cost and time of analysis [60]. The application of the HTS approach allowing the analysis of MFS associated genes and genes associated with disorders in differential diagnosis may determine a better and rapid definition of the diagnosis and of the genes involved in the clinical manifestations of the patients with important implications for the management of the individuals affected by these complex disorders. Nevertheless, HTS equally requires the presence in the laboratory of expert biologists/biotechnologists/bioinformaticians to perform analysis and mutation pathogenicity evaluation.

## 5. Conclusions and Take-Home Messages

Our patient successfully underwent valve-sparing aortic surgery in 2008, by the replacement with a prosthetic tube of the aortic root and of the ascending aorta. Four months after the intervention, the patient experienced a paroxysmal atrial fibrillation which was effectively cardioverted electrically. Since then, the patient is controlled on a yearly basis. In 2011, transthoracic echocardiography detected moderate aortic insufficiency and an MRI study showed the satisfactory outcome of the surgical intervention on the aorta, excluding the recurrence of any further dilatation in other aortic regions. Moreover, both echocardiographic and MRI findings were stable at last evaluation in February 2016, confirming the persistence of the results of surgery.

After surgery, the follow-up strategy of a patient affected by an aortic aneurysm resembles that used before aortic repair, with differences in terms of frequency according to the pathology and type of surgery (i.e., more frequent controls for patients undergoing urgent surgery for dissection) [15]. Targets of clinical and therapeutic approaches are the same as those utilized before surgical intervention. In MFS, in particular, prosecution of *β*-blockers therapy and, possibly, of TGF-*β*-antagonism by ARB is recommended (unless contraindicated). Transthoracic echocardiography is effective in these patients for assessing the anatomy of the root aortic prosthesis and the function of both the aortic and mitral valve. Nonetheless, due to chest deformity, or other causes of poor acoustic windows, echocardiography may not be adequate in all the patients. Moreover, progressive aortic dilatation can occur in other regions of the thoracic and abdominal aorta, which are not amenable to ultrasound scanning. Thus, periodic assessment with MR or CT scan of the entire thoracoabdominal aorta is needed, with interval between controls adjusted based absolute aortic size and progression of the disease.

In conclusion, this case report demonstrates how family history together with a comprehensive clinical and genetic evaluation of patients with TAAD is necessary for clinical diagnosis in order to achieve a documented differential diagnosis aimed at tailoring follow-up and therapeutic medical and surgical strategies which may be noticeably different based on different etiologies.

Such an approach requires a tertiary referral center in order to offer a multidisciplinary assessment (to study the systemic components defining either syndromic or nonsyndromic conditions), a coordinated multimodal imaging approach, biomolecular and genetic skills, and, in selected cases, surgical (pre- or postintervention) evaluation.

The need of genetic counseling should be strongly reminded both before and after mutation screening analysis in the center in which the analysis has been performed. The genetic counseling is required independently of the purpose of counseling itself (i.e., definition of diagnosis in potential MFS, future prenatal diagnosis, and early diagnosis).

## Figures and Tables

**Table 1:** Nonsyndromic and main syndromic disorders associated with thoracic aortic aneurysm.

Classification	Frequency	Gene name	Gene symbol	Inher.
*Nonsyndromic*				
Aortic aneurysm, familial thoracic 1 (AAT1)	Rare	—	*Chr.11q23.3-q24*	AD
Aortic aneurysm, familial thoracic 1 (AAT2)	Rare	—	*Chr.5q13-q14*	AD
Aortic aneurysm, familial thoracic 3 (AAT3)	3% of TAA	Transforming growth factor-beta receptor, type II	*TGFBR2*	AD
Aortic aneurysm, familial thoracic 4 (AAT4)	1-2% of TAA	Myosin, heavy chain 11, smooth muscle	*MYH11*	AD
Aortic aneurysm, familial thoracic 5 (AAT5)	2% of TAA	Transforming growth factor-beta receptor, type I	*TGFBR1*	AD
Aortic aneurysm, familial thoracic 6 (AAT6)	10–15% of TAA	Actin, alpha-2, smooth muscle, and aorta	*ACTA2*	AD
Aortic aneurysm, familial thoracic 7 (AAT7)	1% of TAA	Myosin light chain kinase	*MYLK*	AD
Aortic aneurysm, familial thoracic 7 (AAT8)	Rare	Protein kinase, cGMP-dependent, regulatory, and type I	*PRKG1*	AD

*Syndromic*				
Marfan syndrome	1 : 5,000–10,000	Fibrillin 1	*FBN1*	AD
Loeys-Dietz syndrome 1	Rare	Transforming growth factor-beta receptor, type I	*TGFBR1*	AD
Loeys-Dietz syndrome 2	Rare	Transforming growth factor-beta receptor, type II	*TGFBR2*	AD
Loeys-Dietz syndrome 3 or aneurysm osteoarthritis syndrome	Rare	Mothers against decapentaplegic homolog 3	*SMAD3*	AD
Loeys-Dietz syndrome 4	Rare	Transforming growth factor-beta 2	*TGFB2*	AD
Loeys-Dietz syndrome 5	Rare	Transforming growth factor-beta 3	*TGFB3*	AD
Vascular Ehlers-Danlos syndrome	1 : 100,000	Collagen, type III, alpha-1	*COL3A1*	AD
Arterial tortuosity syndrome	Rare	Solute carrier family 2 (facilitated glucose transporter), member 10	*SLC2A10*	AR

**Table 2:** Revised Ghent criteria for Marfan syndrome diagnosis (modified from Loeys et al. 2010) [11].

*In the absence of family history of MFS*	
(i) Aortic dilatation^*∗*^ (*Z*-score ≥ 2) *and* Ectopia lentis = MFS	
(ii) Aortic dilatation^*∗*^ (*Z*-score ≥ 2) *and FBN1 *mutation^*∗∗*^ = MFS	
(iii) Aortic dilatation^*∗*^ (*Z*-score ≥ 2) *and* systemic score ≥ 7 points (Table 3) = MFS^§^	
(iv) Ectopia lentis *and FBN1* mutation with known aortic dilatation^∧^ = MFS	

*In the presence of family history of MFS*	
(i) Ectopia lentis *and* familial history of MFS = MFS	
(ii) Systemic score ≥ 7 points (Table 3) *and* familial history of MFS = MFS^§^	
(iii) Aortic dilatation^*∗*^ (*Z*-score ≥ 2 above 20 years old, ≥3 below 20 years) + familial history of MFS = MFS^§^	

*∗*: aortic diameter at the sinuses of valsalva above indicated *Z*-score or aortic root dissection.

^*∗∗*^FBN1 (fibrillin 1) mutation is defined according to the following criteria:

(i) Mutation previously shown to segregate in Marfan family.

(ii) *De novo* (with proven paternity and absence of disease in parents) mutation belonging to one of the five following categories:

(1) nonsense mutation,

(2) in frame and out of frame deletion/insertion,

(3) splice site mutations affecting canonical splice sequence or shown to alter splicing on mRNA/cDNA level,

(4) missense affecting/creating cysteine residues,

(5) missense affecting conserved residues of the EGF consensus sequence ((D/N)*X*(D/N)(E/Q)*X*_*m*_(D/N)*X*_*n*_(Y/F) with *m* and *n* representing variable number of residues; D aspartic acid, N asparagine, E glutamic acid, Q glutamine, Y tyrosine, and F phenylalanine).

(iii) Other missense mutations: segregation in family if possible and absence in 400 ethnically matched control chromosomes, if no, family history absence in 400 ethnically matched control chromosomes.

(iv) Linkage of haplotype for *n* ≥ 6 meioses to the *FBN1* locus.

^∧^ *FBN1* mutation that has been identified in an individual with aortic aneurysm.

^§^Caveat: without discriminating features of Sphrintzen-Goldberg syndrome, Loeys-Dietz syndrome, or vascular form of Ehlers-Danlos syndrome *and* after *TGFBR1/2*, collagen biochemistry, and *COL3A1* testing if indicated.

**Table 3:** Manifestations and signs included in systemic score and systemic score calculation: maximum score = 20 points; score ≥ 7: systemic involvement [11].

Manifestations and signs	Score
Wrist *and* thumb sign	**3 **(wrist or thumb sign: **1**)
Pectus carinatum deformity	**2 **(pectus excavatum or chest asymmetry: **1**)
Hindfoot deformity	**2 **(plain pes planus: **1**)
Pneumothorax	**2**
Dural ectasia	**2**
Protrusio acetabuli	**2**
Reduced US/LS *and* increased arm/height *and* no severe scoliosis	**1**
Scoliosis or thoracolumbar kyphosis	**1**
Reduced elbow extension	**1**
Facial features (3/5)	**1 **(dolichocephaly, enophthalmos, downslanting palpebral fissures, malar hypoplasia, and retrognathia)
Skin striae	**1**
Myopia > 3 diopters	**1**
Mitral valve prolapse (all types)	**1**
